# Relatively Low HIV Infection Rates in Rural Uganda, but with High Potential for a Rise: A Cohort Study in Kayunga District, Uganda

**DOI:** 10.1371/journal.pone.0004145

**Published:** 2009-01-07

**Authors:** David Guwatudde, Fred Wabwire-Mangen, Leigh Anne Eller, Michael Eller, Francine McCutchan, Hannah Kibuuka, Monica Millard, Nelson Sewankambo, David Serwadda, Nelson Michael, Merlin Robb, the Kayunga Cohort Research Team

**Affiliations:** 1 Makerere University-Walter Reed Project, Kampala, Uganda; 2 Makerere University School of Public Health, Kampala, Uganda; 3 The Henry M. Jackson Foundation, Rockville, Maryland, United States of America; 4 Makerere University School of Medicine, Kampala, Uganda; 5 Division of Retrovirology, Walter Reed Army Institute of Research, Rockville, Maryland, United States of America; University of Cape Town, South Africa

## Abstract

**Background:**

Few studies have been conducted in Uganda to identify and quantify the determinants of HIV-1 infection. We report results from a community-based cohort study, whose primary objectives were to determine HIV-1 prevalence, incidence, and determinants of these infections, among other objectives.

**Methodology:**

Consenting volunteers from the rural district of Kayunga in Uganda aged 15–49 years were enrolled between March and July 2006. Participants were evaluated every six months. A questionnaire that collected information on behavioral and other HIV-1 risk factors was administered, and a blood sample obtained for laboratory analysis at each study visit.

**Principal Findings:**

HIV-1 prevalence among the 2025 participants was 9.9% (95% CI = 8.6%–11.2%). By the end of 12 months of follow-up, 1689.7 person-years had been accumulated, with a median follow-up time of 11.97 months. Thirteen HIV-1 incident cases were detected giving an annual HIV-1 incidence of 0.77% (95% CI = 0.35–1.19). Prevalence of HSV-2 infection was 57% and was strongly associated with prevalent HIV-1 infection (adjusted Odds Ratio = 3.9, 95% CI = 2.50–6.17); as well as incident HIV-1 infection (adjusted Rate Ratio (RR) = 8.7, 95% CI = 1.11–67.2). The single most important behavioral characteristic associated with incident HIV infection was the number of times in the past 6 months, a participant had sex with person(s) they suspected/knew were having sex with others; attaining statistical significance at 10 times and higher (adjusted RR = 6.3, 95% CI = 1.73–23.1). By the end of 12 months of follow-up, 259 participants (13%) were lost to follow-up, 13 (0.6%) had died, and 2 (0.1%) had withdrawn consent.

**Conclusions:**

Despite relatively low HIV-1 incidence observed in this community, prevalence remains relatively high. In the presence of high prevalence of HSV-2 infection and the behavioral characteristic of having sex with more than one partner, there is potential for increase in HIV-1 incidence.

## Introduction

Accurate estimates of HIV-1 infection rates (prevalence and incidence), and identification of associated risk factors in a population is important for determining appropriate interventions. Few community-based studies have been conducted in Uganda to assess HIV-1 infection rates and determinants of these infections. Previously reported HIV-1 prevalence and incident estimates have primarily been based on sentinel surveillance data at antenatal clinics. Although some validation studies have shown that HIV-1 trends among women attending antenatal clinics provide a fairly accurate reflection of trends in HIV-1 infection among females in the general population, it may not correctly reflect the overall HIV-1 infection rates in the general population as HIV-1 infection tends to be generally higher among females compared to males [1]. Other validation studies have shown that HIV-1 surveillance based on antenatal clinic data overestimate trends in HIV-1 infection in some age-groups in the general population, whereas in other age-groups it underestimates [2]. Community-based studies therefore provide more accurate estimates of HIV-1 infection rates in the general population.

The few community-based studies that have assessed the distribution and determinants HIV-1 infection in Uganda have mostly been from cohort studies conducted in the south western Ugandan districts of Rakai and Masaka [3]–[6]. Nationally, there have only been two national surveys conducted by Uganda's Ministry of Health (MOH) to estimate HIV-1 prevalence, with the most recent conducted in 2005 [7]. An attempt was made to estimate incidence by subjecting all HIV-1 positive samples collected in the most recent survey to the BED-assay [8]. However, there is evidence suggesting that this test overestimates incidence [9]. Therefore, the distribution and determinants of HIV-1 infection rates in other parts of Uganda, other than the districts of Rakai and Masaka, have not been examined.

In this article the authors report results from a community-based closed cohort study, which is part of preparatory research for Phase III HIV vaccine efficacy trials being conducted by the Makerere University-Walter Reed Project in Uganda, a project sponsored by the US Military HIV Vaccine Research Program network. The study was conducted in Kayunga District, a rural district located in the mid-central part of Uganda, with an estimated population of 320,000 [10]. The district has nine Sub-Counties, and six of these were randomly selected as the study area. The estimated population of individuals aged 15–49 years in the six sub-counties is 111,394 people [10]. The main objectives of the study were to determine HIV-1 prevalence, incidence and risk factors associated with HIV-1 infection, to identify HIV-1 viral subtypes circulating in this part of Uganda, and determine cohort participant retention and willingness to participate in future HIV vaccine efficacy trials.

## Methods

### Ethics Statement

The Institutional Review Boards of Uganda's National Council for Science and Technology (UNCST), that is, the National AIDS Research Committee (NARC); and that of the Walter Reed Army Institute of Research (WRAIR), approved the conduct of this study, including approval of the written informed consent form used in this study.

### Recruitment

Recruitment for participants was conducted using two approaches; the “house-to-house” recruitment strategy, and the “broadcast” recruitment strategy. The house-to-house recruitment strategy involved trained Community Mobilizers (CMs) moving from house-to-house providing information about the study to household members. The CMs further invited household members to information seminars that were organized by the research team at strategic locations within the community. At the end of each information seminar, individuals aged 15–49 years were encouraged to report to a designated health center within their community, at which the research team provided more details about participation in the cohort study. One health center within each of the six study sub-counties was selected to serve as the study site for enrolling and follow-up evaluations of participants.

The “broadcast” recruitment strategy involved use of IRB-approved banners, fliers, posters and public announcements in the urban section of Kayunga town, the main town of the district. The messages invited potential volunteers to the health center nearest to Kayunga town to be provided more details about participation in the study.

### Enrollment and study procedures

At the designated health centers, the study was explained further in a one-on-one session with a study counselor and a written informed consent obtained. Residents of the six study sub-counties aged 15 to 49 years, willing to provide a blood sample and able to give consent (plus assent for volunteers aged 15 to 17 years) were enrolled. Contact information was then obtained, a blood sample collected, and a questionnaire administered. The questionnaire collected information on basic social demographics, general health, knowledge about vaccines, willingness to participate in a future preventive HIV-1 vaccine efficacy trials, HIV/AIDS knowledge, and behavioral risk factors for HIV-1. Data collected on behavioral risk factors for HIV included; type and number of sexual partners in past 6 months, frequency of having sex with such partners, frequency of condom use, having sex after taking alcohol, paying or being paid for sex, etc. Participants also provided a medical history and received a physical examination that included observations for weight, temperature, blood pressure, pulse and presence of lymphadenopathy. A blood sample was then obtained for HIV-1 testing and other laboratory study tests. Participants were evaluated every six months and at each study visit trained interviewers administered a standardized questionnaire.

**Figure 1:**
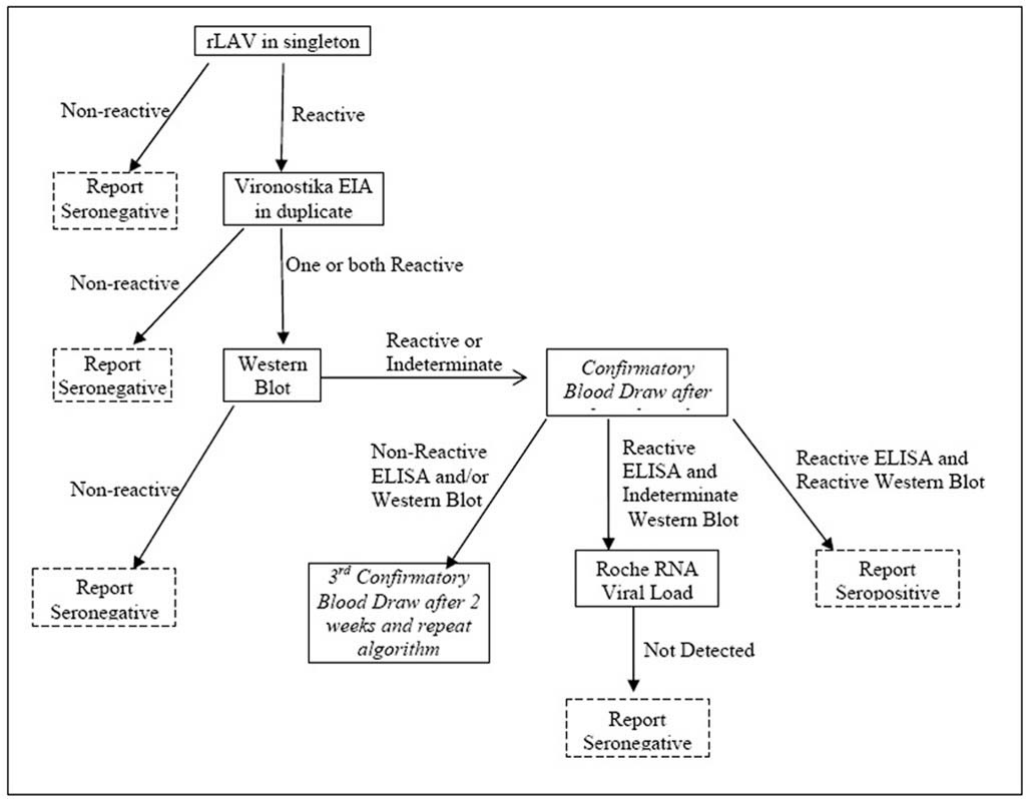
Algorithm for HIV testing.

HIV testing was performed on all participants, and at each study visit. Testing was performed using previously described ELISA and western blot methods [11]. Samples found to be indeternminate or reactive in the Western Blot were subjected to the Amplicor HIV-1 Monitor test, version 1.5 (Roche Dignostics, Indianapolis, Ind.) in the standard mode. The algorithm for HIV-1 testing and sero-status classification is summarized in Figure 1. All identified HIV-1 sero-positive participants' plasma samples were tested for virus subtype using previously described multi-region hybridization assay (MHA), version 2, for HIV-1 subtypes A, C, D, recombinants, and dual infections [3], [12], [13].

HIV pre-test and post-test counseling sessions were conducted in a private room with great attention paid to the preservation of participant confidentiality. Participants returned to receive their blood test results two weeks after the blood draw. Consistent with Ugandan guidelines, test results including HIV results were provided to participants only if they were ready to receive their results. In case a participant was not ready to receive their results, continued post test counseling would be provided at subsequent study visits. Identified HIV sero-positive participants were referred to Kayunga District Hospital where they would receive comprehensive HIV care and treatment.

Screening for Hepatitis B surface antigen (HBsAg) was performed using the Genetic Systems HBsAg EIA 3.0 (BioRad Laboratories, Redmond, WA). Repeatedly reactive samples were confirmed using the Genetic Systems Confirmatory Assay 3.0 (BioRad Laboratories, Redmond, WA). Screening for anti-Hepatitis C antibody was performed using the Ortho HCV Version 3.0 ELISA Tests System. Repeatedly reactive samples were tested in the Chiron RIBA HCV 3.0 SIA (Chiron Corporation, Emeryville, CA). Syphilis serology was conducted using the Wampole Impact Rapid Plasma Reagin (RPR) kit (Inverness Medical Professional Diagnostics, Princeton, NJ) and confirmed using a Serodia Treponema pallidum particle agglutination (TP-PA) test (Fujirebio, Tokyo, Japan). All samples were tested for the Herpes Simplex Virus Type 2 (HSV-2) antibody with an ELISA kit from Focus Diagnostics (Focus Technologies, Cypres, Calif.). A higher sample index value cutoff of 3.4 was used to opyimize the performance of the assay as previously described [14].

### Participant Follow-up and Retention Strategies

In order to minimize loss to follow-up, CMs maintained contact with study participants by making a visit to their home mid-way between, and one week before the scheduled 6–monthly visit reminding each participant of their scheduled follow-up visit. Further, if a study participant purportedly away from their place of residence in between their study visits, attempts were made to trace them to their new locations using the provided contact information.

Participants were treated for acute febrile infections and minor ailments free of charge at any time during the study period. In case of a more severe disease that the study medical staff could not treat as outpatient care, participants were referred to Kayunga District Hospital where they could be hospitalized during treatment and care.

### Statistical Analysis

HIV-1 prevalence was calculated as the percentage of participants enrolled into the study, who tested HIV-1 positive at baseline. HIV-1 incidence rates were calculated using person-time analysis, with the numerator being the number of participants who sero-converted during follow-up, the denominator being the cumulative number of person-years contributed by the HIV-1 sero-negative participants, from baseline onwards during follow-up. HIV-1 sero-negative individuals contributed person-time to the denominator beginning from baseline until the time of sero-conversion or until conclusion of the study or when censored. For HIV-1 sero-converters, the date of sero-conversion was calculated as the mid-time point between the visit date of the last HIV-1 sero-negative test and first HIV-1 sero-positive test. All HIV-1 sero-converters contributed zero person-time from their calculated date of sero-conversion onwards.

Logistic regression analysis was used to identify factors associated with prevalent HIV-1 infection. Odds ratios (OR) with their corresponding 95% confidence intervals (95% CI) are reported. Poisson regression analysis was used to identify factors associated new HIV infections by comparing incidence rates of HIV-1 infection between participants with the baseline factor of interest and those without. Incidence rate ratios (RR) with their corresponding 95% CI are reported. All risk factors variables for HIV infection measured in this study were examined for possible association with prevalent, and/or incident HIV-1 infection. Except for a few selected variables, only variables found to be significantly associated with prevalent or incident HIV-1 infection are presented. All statistical analyses were performed using the Statistical Analysis Software Version 8.2 [15].

## Results

### Characteristics of cohort participants

A total of 2025 participants, representing 1.8% of community members aged 15–49 years within the study area were enrolled, of which 945 (47%) were males. The average age of the participants was 28.5 (standard deviation = 8.1) years. Male participants were on average younger with an average age of 27.3 (±8.0), compared to females with an average age of 28.6 (±8.2), *p-value* = 0.004. The education level of participants was low as the majority had at most completed primary school level (53.0%). The main occupation of the participants was farming (49.4%). Table 1 summarizes the baseline demographic and social characteristics of the participants.

**Table 1:** Baseline demographic and social characteristics of participants.

Characteristic	Category	- n-	Summary measure
All participants	-	2025	-
Sex:	Males	945	46.7%
	Females	1080	53.3%
Age (yrs):	15–19	283	14.0%
	20–24	451	22.3%
	25–29	354	17.5%
	30–34	370	18.3%
	35–39	344	17.0%
	40–44	158	7.8%
	45–49	65	3.2%
	Mean±SD	-	28.5±8.1
Employment category:	Farmer	1000	49.4%
	Student	288	14.2%
	Trader	164	8.1%
	Unemployed	101	5.0%
	Civil servant/NGO employee	63	3.1%
	Housewife	48	2.4%
	Bar/Restaurant worker	16	0.8%
	Others	345	17.0%
Education level completed:	None	148	7.3%
	Primary unfinished	652	32.2%
	Primary finished	274	13.5%
	O'level unfinished	580	28.6%
	O'level finished	183	9.0%
	Higher than O'level	188	9.3%
Marital status:	Single	574	28.3%
	Married to 1 spouse	1091	53.9%
	Married to more than 1 spouse	62	3.1%
	Cohabiting	32	1.6%
	Separated/Divorced	254	12.5%
	Widowed	12	0.6%
Sub-county of residence:	Kayunga Town	281	46.0%
	Kayunga	931	13.9%
	Kayonza	262	12.9%
	Kangulumira	257	12.7%
	Nazigo	229	11.3%
	Bbaale	65	3.2%

### Prevalence of HIV infection

A total of 201 participants tested HIV-1 sero-positive at baseline, giving an overall prevalence of 9.9% (95% CI = 8.6%–11.2%), with significantly higher prevalence among females, 12.5% versus 7% among males (*p-value*<0.001). Across the different age groups, HIV-1 prevalence was lowest among participants aged 15–24 years (5.8%) and highest among participants aged 40–44 years (17.7%). On average HIV-1 prevalence increased with age with an overall adjusted OR of 1.1 (95% CI = 1.04–1.08) per year.

A total of 1155 (57%) tested positive for HSV-2 infection, with significantly higher prevalence among females, 66.3% versus 46.5% among males (*p-value*<0.001). HSV2 infection was significantly associated with prevalent HIV-1 infections with an adjusted OR of 3.9, 95% CI = 2.50–6.17. Of the 223 participants who tested positive for syphilis infection, 44 (19.7%) were more likely to test HIV-1 sero-positive compared to those testing negative for syphilis 157 (8.7%), with an adjusted OR = 2.1 [1.42–3.16]. Participants reporting to ever have had a traditional skin cutting on their body were more likely to test HIV positive with a prevalence of 14.6% versus those without a traditional skin cutting 6.9% (*p-value*<0.001), with an adjusted OR = 1.7 (95% CI = 1.28–2.40). Among male participants who reported to have ever had sex, HIV prevalence was 5.7% among the circumcised versus 8.4% among the un-circumcised, but this difference was not statistically significant, adjusted OR 0.6 (95% CI = 0.34–1.16). Among the 491 participants reporting to have had sex with a casual partner in the past 6 months, frequency of condom use was not significantly associated with prevalent HIV-1 infection (*p-value* = *0.984*).

Of the 513 participants reporting to have had sex in the past 6 months with persons they suspected/knew to be having sex with other people, 240 (46.8%) never used a condom, 67 (13.1%) used a condom a few times, 52 (10.1%) used a condom half of the time, 37 (7.2%) used a condom most of the time, and 117 (22.8%) always used a condom. Condom use in this group was also not significantly associated with prevalent HIV-1 infection (*p-value* = *0.806*). Table 2 summarizes the distribution of HIV prevalence, and multi-variable analysis results of selected baseline characteristics.

**Table 2:** Prevalence of HIV infection, by baseline characteristics of participants.

Characteristic	Category	- n-	# HIV +ve (%)	Adjusted OR	95% CI	p-value
All participants	-	2025	201 (9.9)	-	-	-
Sex:	Males	945	66 (7.0)	1.0		-
	Females	1080	135 (12.5)	1.0	0.72–1.43	0.933
Age (yrs):	15–24	734	27 (5.8)	1.0	-	-
	25–29	354	34 (9.6)	1.5	0.87–2.57	0.734
	30–34	370	55 (14.9)	2.2	1.34–3.63	0.006
	35–39	344	49 (14.2)	1.7	1.04–2.91	0.207
	40–44	158	28 (17.7)	1.9	1.03–3.39	0.167
	45–49	65	6 (9.2)	0.7	0.27–1.92	0.082
Marital status:	Single or Married	1726	124 (9.4)		1.0	-
	Cohabiting	32	8 (25.0)	4.1	1.74–9.78	0.075
	Separated/Divorced	267	69 (25.8)	3.6	2.53–5.11	0.032
Circumcision status of males who have ever had sex (n = 850):	No	536	45 (8.4)	1.0	-	-
	Yes	314	18 (5.7)	0.6	0.34–1.16	0.137
Ever had a traditional skin cutting on body?:	No	1246	86 (6.9)	1.0		-
	Yes	779	114 (14.6)	1.7	1.28–2.40	<0.001
HSV2 infection:	No	870	25 (2.9)	1.0		-
	Yes	1155	176 (15.2)	3.9	2.50–6.17	<0.001
Syphilis infection:	No	1802	157 (8.7)	1.0		-
	Yes	223	44 (19.7)	2.1	1.42–3.16	<0.001
Frequency of condom use among participants who had sex with a casual partner in past 6 months (n = 419)	Every time	52	16 (7.3)	1.0		
	Never	55	13 (10.9)	0.9	0.38–2.12	0.347
	A few times	46	6 (11.5)	0.9	0.29–2.64	0.522
	Half of the time		10 (18.2)	0.2	0.09–1.64	0.080
	Most times		3 (6.5)	0.7	0.17–2.61	0.984
# of times in past 6 months had sex with someone thought to be having sex with others:	None	1520	154 (10.1)	1.0		
	1–2 times	171	13 (7.6)			
	3–10 times	178	13 (7.3)	0.5	0.26–0.89	0.097
	>10 times	156	21 (13.5)	1.1	0.62–1.79	0.085

### Incidence of HIV Infection

By the end of 12 months of follow-up, a total of 1689.7 person-years had been accumulated among the 1824 HIV-1 sero-negative participants, with a median follow-up time of 11.97 months (Inter-Quartile Range of 11.96 to 11.97 months). Thirthteen participants sero-converted during follow-up, giving an estimated incidence of 0.77 per 100 person-years, (95% CI = 0.35–1.19). HSV-2 infection at baseline was strongly associated with HIV-1 incidence with an adjusted RR of 8.7 (95% CI = 1.11–67.2). Additionally, participants reporting to have had sex in the past 6 months with a partner(s) they knew or suspected of having sex with others was strongly associated with HIV-1 incidence. This risk increased with the number of coital acts in the past 6 months, attaining statistical significance at 10 times or more with an adjusted RR of 6.3 (95% CI = 1.73–23.1).

Annual HIV-1 incidence was highest in the age group 25–29 years (1.68%), followed by the 35–39 years age group (1.05%). However the differences between age groups were not statistically significant (see Table 3). Of the 13 HIV-1incident cases, 9 were females with an estimated annual incidence of 1.02%, compared to 4 cases among males with an estimated annual incidence of 0.49%. The difference between male and female participants did not attain statistical significance (adjusted RR = 1.5, 95% CI = 0.41–5.14). A significantly higher incidence of HIV-1 infection was observed among participants residing in one of the sub-counties with large fishing communities (Bbaale sub-County), compared to any of the other five study sub-counties. Table 3 summarizes the incidence rate information.

**Table 3:** Association between selected baseline factors to incident HIV infections.

Baseline Characteristic	Category	New cases/person-yrs	Incidence/per 100 person-yrs	Adjusted RR	95% CI	p-value
All participants		13/1689.7	0.77	-	-	-
Sex:	Males	4/809.8	0.49	1.0		
	Females	9/880.0	1.02	1.46	0.41–5.14	0.556
Age (yrs):	15–19	0/251.9	-		-	
	20–24	2/372.2	0.54	1.0		
	25–29	5/297.9	1.68	1.41	0.26–7.53	0.689
	30–34	3/302.5	0.99	0.67	0.11–4.15	0.667
	35–39	3/282.4	1.06	0.58	0.09–3.68	0.568
	40–44	0/126.1	-		-	
	45–49	0/56.9	-		-	
Sub-county of residence:	Kayunga Town	2/748.6	0.27	1.0		
	Bbaale	3/50.6	5.93	25.7	4.18–158.1	<0.001
	Kangulumira	2/226.0	0.88	3.56	0.50–25.4	0.205
	Kayonza	1/237.6	0.42	1.10	0.10–12.2	0.935
	Kayunga	3/227.2	1.32	4.50	0.75–27.1	0.100
	Nazigo	2/199.6	1.00	4.27	0.60–30.4	0.147
Marital status:	Never married	3/493.8	0.61	1.0		-
	Married, 1 spouse	6/942.9	0.64	0.18	0.04–0.87	0.033
	Married, >1 spouse	0/53.1	-		-	-
	Cohabiting	0/21.3	-		-	-
	Separated/Divorced	4/171.0	2.34	2.12	0.37–12.2	0.838
	Widowed	0/79.5	-		-	-
# of times in past 6 months had sex with someone thought to be having sex with others:	None	6/1094.0	0.55	1.0		
	1–2 times	1/148.0	0.68	0.83	0.10–7.01	0.863
	3–10 times	2/152.8	1.31	2.11	0.42–10.5	0.361
	>10 times	4/128.9	3.10	6.33	1.73–23.1	0.005
HSV2 infection:	No	1/771.8	0.13	1.0		
	Yes	12/908.9	1.32	8.65	1.11–67.2	0.039

By the end of 12 months of follow-up, 13 participants (0.6%) were known to have died, 2 (0.1%) had withdrawn consent from continuing participation in the study, 259 (13%) had been lost to follow-up, while the remaining 1751 were still in active follow-up. Thus cohort retention by the end of 12 months of follow-up was 86%. Loss to follow-up was highest in the age groups less than 25 years with an average of 21.4%, compared to those aged 25 years or older with an average of 7.9% (*p-value*<0.001).

### Other Results

MHA analysis was performed on 105 of the 201 participants testing HIV-1 positive at baseline. The predominant HIV-1 subtype was clade A (45%), followed by clade D (23%), and clade C (1%). The rest were recombinant HIV-1 sub-types (23%), or dually reactive (8%). Prevalence of Hepatitis B surface antigen was 4.6%, for Hepatitis C antibody it was 0.6%, and for Syphilis it was 11.0%.

## Discussion

Results from this study show a relatively high HIV-1 prevalence of 9.9% in this rural Ugandan community, compared to the national average of 6.4% [7]. But the annual HIV-1 incidence of 0.77% is lower than what has been reported from similar rural Ugandan communities of Rakai and Masaka districts that has ranged between 1.03 to 1.50% per year [3], [16]–[19]. A number of studies have shown a decline in Ugandan HIV-1 infection rates [7], [20]–[22]. For Kayunga District, until 2005 there had been few HIV-1 interventions compared to other parts of Uganda that have benefited from intensified HIV-1 prevention programs for longer periods of time, which may explain the relatively higher HIV-1 prevalence in this community. Conversely, the relatively low HIV-1 incidence gives optimism that the burden of HIV-1 infection will decline in the near future, if the current HIV-1 prevention interventions are continued.

Our study also shows that the burden of HIV-1 infection is concentrated in the age groups above 30 years. This distribution is consistent with findings from other studies showing a shift in the burden of HIV-1 infection from young adults aged 20–25 years old, as previously described in the late 1980s and early 1990s [23], [24]; to older adults aged 30–40 years [7]. This shift in burden has been attributed to HIV-1 risk reduction among young adults [25]. The relatively low HIV-1 prevalence among young adults compared to older adults seem to suggest that most of the prevalent HIV-1 infections in this community could have occurred in the remote past, suggesting a possible cohort effect.

Six of the 13 HIV-1 incident cases occurred among participants reporting to be married to one spouse. A closer examination of these incident cases revealed that 4 of them reported at baseline to have had sex at least 3 times in the past six months, with a partner they knew or suspected of having sex with others. Some investigators have asserted that a substantial number of new HIV-1 infections in Uganda are now occurring among married individuals engaging in risky sexual behavior outside of their marital relationships [26], [27]. Indeed the single most important behavioral characteristic identified in our study as fueling the spread of HIV-1 infection in this community was having sex with partners known or suspected of having sex with others. Furthermore, there was a clear dose response of this behavior with the increasing number of times one had sex with such partners in the past 6 months. Therefore the message of “being faithful” to one's HIV-1 uninfected faithful sexual partner(s) must be emphasized in this community as part of the HIV-1 risk reduction package.

Our study also shows a high prevalence of HSV-2 infection in this rural community that was strongly associated with prevalent, as well as incident HIV-1 infections. HSV-2 infection has been identified as the most common cause of genital ulcerative disease worldwide [28], [29]. The major public health importance of HSV-2 relates to its potential role in facilitating HIV transmission [29], [30]. Genital ulcerative disease enhances the infectiousness of HIV-1 positive subjects and the susceptibility of HIV-1 uninfected subject's susceptibility to HIV-1 infection. Accumulating data suggest that HSV-2 may be responsible for a substantial proportion of new HIV-1 infections in many parts of Africa [31]–[33]. There is therefore an urgent need to provide health education about HSV-2 infection and its potential role in enhancing HIV-1 transmission.

The predominant HIV-1 sub-types identified in this study was clade A (45%), followed by clade D (23%). The distribution of HIV-1 subtypes found in this community is slightly different from that found in another Ugandan rural district of Rakai, located in the southern western part of the country, where clade D was the most predominant (54%), followed by clade A (15%) [34]. The distribution in our study is however similar to that found in a number of studies conducted in neighboring Kenya where clade A has been found to be predominant (greater than 50% in all studies), followed by clade D [35], [36].

One of the possible limitations in this analysis is that the follow-up period may not have been long enough to accumulate enough person-time to enable us identify all risk factors for incident HIV-1 infections in this community. The median follow-up time was only 11.89 months. Secondly, it is possible that the HIV-1 risk level of individuals who volunteered to participate in this study is quite different from other community members who did participate in this study, thus creating a selection bias in our study. It is difficult to tell the possible direction of this bias, but we believe that findings from this study do not differ significantly from the truth, and therefore provide useful information.

We conclude that although HIV-1 prevalence in this community is still relatively high, incidence is relatively low. The low incidence gives optimism that behaviors are changing and this will lead to further reductions in the HIV-1 infection rates. However, the high prevalence of HIV-1 infection, in presence of high prevalence of HSV-2 infection, and the behavior of concurrently having sex with multiple partners are suitable conditions for a rise in HIV-1 infection rates in the near future. Behavioral change has been identified as one of the most important factors that has significantly contributed to the decline in HIV-1 infection rates in Uganda, especially sexual partner reduction [20], [21], [37]. Behavioral HIV-1 risk reduction interventions should be intensified to enable further reductions in HIV-1 infection rates in this community. Health education about HSV-2 infection should be intensified, especially regarding its potential role in enhancing HIV-1 transmission.
